# Supplementary material for: All-at-once RNA folding with 3D motif prediction framed by evolutionary information
Source: Nat Methods. 2025 Oct 3;22(10):2094–106. doi: 10.1038/s41592-025-02833-w (PMC12510878; doi:10.1038/s41592-025-02833-w)
Supplement: Supplementary file 3 — Source code to run CaCoFold-R3D. Data and results. [file 41592_2025_2833_MOESM3_ESM.gz › supplemental_material/results/CaCoFold-R3D/CaCoFold-R3D_Rfam_vshuffle/RF02120_FTX_2.R2R.sto.pdf]

RF02120_FTX_2

5′–C U G A R A R ● R Y Y Y ○ Y Y C U C U U C U R Y U R Y A Y R ● U C A C U C A C R U G G A A R A U C U G G U R R A C U C U ○ ○ A A A A Y R C U U U U C A A A R A U A U C Y U U C A Y A R U Y C A G C C
